# Tibiofemoral joint structural change from 2.5 to 4.5 years following ACL reconstruction with and without combined meniscal pathology

**DOI:** 10.1186/s12891-019-2687-9

**Published:** 2019-07-04

**Authors:** Xinyang Wang, Kim L. Bennell, Yuanyuan Wang, Tim V. Wrigley, Ans Van Ginckel, Karine Fortin, David J. Saxby, Flavia M. Cicuttini, David G. Lloyd, Christopher J. Vertullo, Julian A. Feller, Tim Whitehead, Price Gallie, Adam L. Bryant

**Affiliations:** 1 Centre for Health, Exercise and Sports Medicine, Department of Physiotherapy, School of Health Sciences, The University of Melbourne, Melbourne, Victoria Australia; 2 Department of Orthopaedic Surgery, Beijing Chao-Yang Hospital, Beijing, China; 3 School of Public Health & Preventive Medicine, Monash University, Alfred Hospital, Melbourne, Victoria Australia; 4 Department of Rehabilitation Sciences and Physiotherapy, Faculty of Medicine and Health Sciences, Ghent University, Ghent, Belgium; 5 School of Allied Health Sciences, Griffith University, Gold Coast, Australia; 6 Core Group for Innovation in Health Technology, Menzies Health Institute Queensland, Gold Coast, Australia; 7 Gold Coast Orthopaedic Research and Education Alliance, Gold Coast, Australia; 8 Knee Research Australia, Gold Coast, Australia; 9 OrthoSport Victoria, Melbourne, Australia; 10 College of Science, Health and Engineering, La Trobe University, Melbourne, Australia; 11 Coast Orthopaedics, Gold Coast, Australia

**Keywords:** Anterior cruciate ligament reconstruction; post-traumatic osteoarthritis; magnetic resonance imaging, Cartilage volume, Cartilage defect, Bone marrow lesions

## Abstract

**Background:**

People who have had anterior cruciate ligament reconstruction (ACLR) are at a high risk of developing tibiofemoral joint (TFJ) osteoarthritis (OA), with concomitant meniscal injury elevating this risk. This study aimed to investigate OA-related morphological change over 2 years in the TFJ among individuals who have undergone ACLR with or without concomitant meniscal pathology and in healthy controls. A secondary aim was to examine associations of baseline TFJ cartilage defects and bone marrow lesions (BML) scores with tibial cartilage volume change in ACLR groups.

**Methods:**

Fifty seven ACLR participants aged 18–40 years (32 isolated ACLR, 25 combined meniscal pathology) underwent knee magnetic resonance imaging (MRI) 2.5 and 4.5 years post-surgery. Nine healthy controls underwent knee MRI at the ~ 2-year intervals. Tibial cartilage volume, TFJ cartilage defects and BMLs were assessed from MRI.

**Results:**

For both ACLR groups, medial and lateral tibial cartilage volume increased over 2 years (*P* <  0.05). Isolated ACLR group had greater annual percentage increase in lateral tibial cartilage volume compared with controls and with the combined group (*P* = 0.03). Cartilage defects remained unchanged across groups. Both ACLR groups showed more lateral tibia BML regression compared with controls (*P* = 0.04). Baseline cartilage defects score was positively associated with cartilage volume increase at lateral tibia (*P* = 0.002) while baseline BMLs score was inversely related to medial tibia cartilage volume increase (*P* = 0.001) in the pooled ACLR group.

**Conclusions:**

Tibial cartilage hypertrophy was apparent in ACLR knees from 2.5 to 4.5 years post-surgery and was partly dependent upon meniscal status together with the nature and location of the underlying pathology at baseline. Magnitude and direction of change in joint pathologies (i.e., cartilage defects, BMLs) were less predictable and either remained stable or improved over follow-up.

**Electronic supplementary material:**

The online version of this article (10.1186/s12891-019-2687-9) contains supplementary material, which is available to authorized users.

## Background

Anterior cruciate ligament (ACL) injury is a common knee injury that primarily affects young, active individuals. ACL reconstruction (ACLR) is the preferred treatment for ACL deficiency, producing favourable short-term outcomes with respect to knee function [1]. However, ACLR does not protect against knee structural degeneration and at 10 years post-ACLR, around 30% of individuals exhibit tibiofemoral joint (TFJ) osteoarthritis (OA) [2–4]. Moreover, concomitant meniscal injury elevates the prevalence of TFJ OA by 2–4 times compared to knees with isolated ACL injury [3, 5, 6]. While the mechanisms underpinning the development of subsequent TFJ OA are not completely clear, residual instability, altered joint-loading patterns and inflammatory processes are likely contributors [7–9]. Assessment of early structural changes in the knee may help clarify the pathophysiology of post-traumatic OA and identify risk factors for disease onset and progression.

Magnetic resonance imaging (MRI) provides a sensitive and non-invasive method for assessing knee morphology including cartilage volume, cartilage defects and bone marrow lesions (BMLs) [10, 11]. Longitudinal studies have revealed hypertrophic swelling-related [12] increases in TFJ cartilage volume and/or thickness from 1 to 5 years following ACL injury, with these being more pronounced in the medial compartment [12–15]. Importantly, cartilage swelling has been reported as a precursor to cartilage loss in early OA populations [16, 17]. Cartilage defects represent early pathology following joint injury, and can be assessed on MRI using semi-quantitative rating scales [18, 19]. Although cartilage degeneration is the central pathology in OA, subchondral BMLs also play an important role in the pathogenesis [20, 21]. BMLs after the ACL injury are frequently seen as a ‘footprint’ of the initial trauma, but exhibit a changeable natural history thereafter [14, 22–24]. The majority of BMLs in the lateral TFJ compartment (50–95%) resolve between one to 6 years following ACLR; however, progression or newly developed BMLs occurred in 25–35% of patients at 2–3 years post-surgery [14, 22–24]. BMLs in the medial TFJ were reported in 0–10% of ACLR patients, and the resolution at 2–3 years ranged from 0 to 100% given the small number at baseline [25, 26]. The clinical relevance of cartilage defects and BMLs has been established in knee OA populations, where both cartilage defects [27, 28] and BMLs [29, 30] have been associated with subsequent cartilage volume loss. Therefore, it is of interest to determine whether similar relationships exist in ACLR knees with and without concomitant meniscal pathology.

The primary aim of this study was to investigate 2-year change in TFJ morphology (i.e., tibial cartilage volume, tibial and femoral cartilage defects, and BMLs) in participants with: (i) isolated ACLR, (ii) ACLR combined with meniscal pathology, and (iii) healthy controls. It was hypothesised that H_1_): Both ACLR groups would exhibit increases in tibial cartilage volume, progression (worsening) of cartilage defects, and changes in BMLs, whilst the healthy control group would show no structural change; H_2_): The ACLR combined with meniscal pathology group would exhibit greater increases in tibial cartilage volume along with greater progression of cartilage defects and BMLs compared with the isolated ACLR group. The secondary aim was to examine whether baseline tibial cartilage defect and BML scores were associated with tibial cartilage volume change over the subsequent 2 years in ACLR patients. It was hypothesised that: H_3_): Higher tibial cartilage defects and BML scores at baseline would be correlated with greater increases in tibial cartilage volume over the subsequent 2 years.

## Methods

Participants were recruited post-operatively in Melbourne (Epworth Hospital) and Gold Coast (Coast Orthopaedics and Orthopaedic Surgery and Sports Medicine Centre) Australia. Inclusion and exclusion criteria have previously been described [31]. Briefly, ACLR participants were aged 18–40 years at 2–3 years post-surgery and had undergone arthroscopically assisted ACLR (within 6 months following an acute ACL tear) using semitendinosus and gracilis (hamstring) tendon autograft. ACLR individuals were excluded if they had: (i) International Cartilage Repair Society (ICRS) cartilage defects grade > 2 at the time of ACLR (ii) other musculoskeletal, cardiovascular or neurological conditions; (iii) previous ACL or subsequent knee surgery on the involved leg; (iv) body mass index (BMI) > 34 kg/m^2^ (to minimise effects of adiposity on gait assessment reliability in the larger study); (v) contraindications to MRI. Eligible participants who had concomitant meniscal pathology (i.e. meniscal injury, repair or partial meniscectomy) at the time of ACLR were assigned to the combined ACLR and meniscal pathology group. Healthy participants (i.e., no prior knee surgery or knee injury) were recruited from associated universities using the following inclusion criteria: i) aged 18–40 years and ii) BMI < 34 kg/m^2^.

### Procedure

All ACLRs were performed by one of the four experienced orthopaedic surgeons using the technique previously outlined [31]. Meniscal lesions were managed with either repair, partial meniscectomy, or no treatment. Partial meniscectomy was performed in participants with meniscal injuries unsuitable for repair where surgeons believed it would be problematic if left untreated. No chondral surgery was undertaken as all lesions were less than ICRS grade 3. An accelerated post-ACLR rehabilitation protocol emphasising rapid restoration of knee range of motion and quadriceps function was prescribed [31].

### Anthropometric and MRI assessments

#### Height, weight, BMI, and sports activity level

Height and weight were measured and used to calculate BMI (kg/m^2^). The sports activity rating scale from the Cincinnati knee rating system was used to assess activity level of the participants [32]. Higher scores indicate higher level of sports participation (0–100).

#### MRI acquisition

MRI scan of the study knee was performed at baseline (~ 2.5 years post-ACLR) and at follow-up 2 years later using whole-body MRI units in Melbourne (3.0 T, Siemens Magnetom Verio, Erlangen, Germany) and Gold Coast (1.5 T, GE Healthcare Signa, Wisconsin, USA). Knees were imaged using T_1_-weighted 3D gradient recall [33] in the sagittal plane and proton density (PD)-weighted fat-saturated spin echo acquisition in the coronal plane. The MRI technical parameters used in Melbourne included: T_1_-weighted, flip angle 10 degrees; repetition time 12.5 ms; echo time 4.9 ms; field of view 16 cm; slice thickness 1.5 mm; 512 × 512 matrix; acquisition time 6 min 58 s; PD-weighted, flip angle 155°, repetition time 2640 msec, echo time 37 msec, slice thickness 3 mm, field of view 16 cm, pixel matrix 256 × 256, acquisition time 1 min 55 s. At Gold Coast, these included: flip angle 55 degrees; repetition time 44 ms; echo time 12 ms; field of view 16 cm; slice thickness 1.5 mm; 256 × 256 matrix; acquisition time 11 min 56 s; PD-weighted, flip angle 155°, repetition time 4000 msec, echo time 50 msec, slice thickness 3 mm, field of view 16 cm, pixel matrix 256 × 256, acquisition time 5 min 26 s [31]. All MRI assessments were blinded to group status.

#### Cartilage volume and bone size

Cartilage volume was measured at the medial and lateral tibia using a validated manual segmentation method [31, 33], by tracing the bone interface and cartilaginous joint surface slice-by-slice on T_1_-weighted images in Osiris (University of Geneva, Switzerland; *Note*: Cartilage volume of the lateral and medial femur was not measured given the difficulties associated with defining and standardizing cartilage boundaries between participants). The intra-rater reliabilities were all above 0.99 (expressed as Intra-class correlation coefficients, ICCs) and inter-rater reliability ranged between 0.98 and 0.99 [31]. Annual percentage change of cartilage volume was calculated for between-group comparisons and was determined by: (cartilage volume at follow-up – cartilage volume at baseline) / cartilage volume at baseline / time between MRI scans in years, presented as percentage. Cross-sectional area of medial and lateral tibial plateaus was measured at baseline for cartilage volume adjustment using the same method as described, and ICCs were between 0.98 and 0.99 [31].

#### Cartilage defects

Cartilage defects were graded at the medial tibia, medial femoral condyle, lateral tibia and lateral femoral condyle using the T_1_-weighted image. The ICRS scoring system were used to assess cartilage defect as previously described: grade 0, normal cartilage; grade 1, focal blistering and intra-cartilaginous low-signal intensity area with an intact surface and base; grade 2, irregularities on the surface or base with loss of thickness < 50%; grade 3, deep ulceration with loss of thickness > 50%; grade 4, full-thickness cartilage wear with exposure of subchondral bone [18, 31]. Intra-observer and inter-observer ICC values ranged between 0.85 and 0.90 [31]. Cartilage defects was defined as ‘progression’ if the score increased by ≥1 (worsened), ‘regression’ if the score decreased by ≥1, or ‘stable’ if the cartilage defect score did not change.

#### Bone marrow lesions

BMLs were examined on the PD-weighted fat-saturated images in the medial tibia, medial femoral condyle, lateral tibia, and lateral femoral condyle. The size of BMLs was graded from 0 to 3 based on the extent of regional involvement in 10 subregions: grade 0, none; grade 1, < 1/3 of the subregional volume; grade 2, 1/3–2/3 of the subregional volume; and grade 3, > 2/3 of the subregional region [31, 34]. The intra- and inter-observer reliability (expressed as weighted kappa-values) in the reading of the BMLs ranged between 0.50 and 1.00. Overall BML scores for each tibial and femoral compartment was determined by identifying the maximum BML score within each of the corresponding compartment subregions. BMLs were defined as ‘progression’ if the score increased by ≥1, ‘regression’ if the score decreased by ≥1 or ‘stable’ if the score did not change over 2 years.

### Statistical analysis

Only participants with all baseline and follow-up measures were included in the main analysis. Characteristics of ACLR and control participants who completed baseline and follow-up assessment were compared with those who did not return for follow-up assessment using independent samples T-tests or Mann-Whitney U tests. Characteristics of the three groups at follow-up were compared using one-way ANOVA or Kruskal-Wallis test. Paired samples T-test and Wilcoxon signed-rank test were used to examine the longitudinal changes in cartilage and subchondral bone parameters within each group. Annual percentage change in cartilage volume was compared between the three groups by analysis of covariance (ANCOVA) adjusting for age, gender, BMI, baseline cartilage defect, and baseline bone size. Chi-squared test and Fisher exact test were used to explore group difference in the change of cartilage defects and BMLs (progression, stable, and regression). In the event of a significant main effect, post hoc comparisons were conducted using the Fisher’s least significant difference (LSD) test or Mann-Whitney U test. The association between predictors (cartilage defect and BML scores) and annual cartilage volume percentage change were examined using univariate and multivariate linear regression with the ACLR groups amalgamated. All statistical analyses were performed using SPSS package (version 22.0, SPSS, Chicago, IBM) with significance accepted at *P* <  0.05. No corrections were made for multiple statistical tests due to the exploratory nature of the research.

## Results

Sixty-six participants from the three groups were tested at baseline and follow-up: i) isolated ACLR (*n* = 32); ii) combined ACLR and meniscal pathology (*n* = 25) and, iii) control (*n* = 9). Characteristics of the three groups are shown in Table 1. In the combined group, 9 participants had a meniscal tear without surgical treatment (4 medial and 5 lateral), 4 had meniscal repair (3 medial r and 1 lateral), and 12 had partial meniscectomy (5 medial and 7 lateral). One participant who had medial meniscectomy also underwent lateral meniscal repair. The combined group exhibited a significantly higher BMI than the isolated ACLR group (*P* = 0.007). The control group had a significantly longer time interval between baseline and follow-up MRI than both surgical groups (*P* < 0.001). No significant differences were found for any other variables. Of those tested at baseline, 57/100 ACLR participants and 9/30 healthy controls returned for follow-up testing. For the ACLR participants, the reason for drop-out included: could not be contacted (*n* = 21); excessive time commitment (*n* = 11); additional ACL or meniscal injury (*n* = 5); MRI scanner problem (n = 2); pregnancy (n = 2); wearing intrauterine device (n = 1) and relocation (n = 1). For the control group, all participants who withdrew from the study due to relocation (*n* = 21). The characteristics of ACLR and control participants who completed follow-up assessment and those who did not were similar, and no significant between-group differences were identified (Additional file 1: Table S1 and Table S2).

**Table 1:** Characteristics of participants

	ACLR isolated (*n* = 32)	ACLR combined (*n* = 25)	Controls (*n* = 9)	*P* value
Age (yr)	30.7 (± 6.4)	30.6 (± 7.1)	28.3 (± 4.0)	0.58
Male, n (%)	19 (59%)	18 (72%)	8 (89%)	0.24
BMI (kg/m^2^)	24.4 (± 3.2)^c^	27.0 (± 3.6)^c^	24.6 (± 3.8)	0.02*
Time from surgery to baseline assessment (yr)	2.5 (± 0.4)	2.5 (± 0.4)	Not applicable	0.92
Time between baseline and follow-up assessments (yr)	2.1 (± 0.2)^a^	2.0 (± 0.2)^b^	2.9 (± 0.4)^a, b^	< 0.001*
Sports activity level at baseline	85 (80, 95)	80 (75, 95)	90 (78, 98)	0.57
Sports activity level at follow-up	85 (80, 95)	80 (65, 95)	90 (78, 98)	0.58

Parametric data presented as mean (± standard deviation), and sports activity levels presented as median (interquartile range). BMI, body mass index. * Significant difference (*P* < 0.05). Post hoc was significantly different for ^a^ isolated ACLR versus controls; ^b^ combined ACLR versus controls; ^c^ isolated ACLR versus combined ACLR.

### Longitudinal within-group changes

Longitudinal change in tibial cartilage volume is shown in Table 2. Both ACLR groups exhibited a significant increase in cartilage volume at both medial and lateral tibia (all *P* < 0.05); while for the control group, a significant increase was found at the medial tibia only (*P* = 0.003).

**Table 2:** Mean (SD) baseline and follow-up tibial cartilage volume (mm^3^) with mean change (95% confidence interval)

Site	ACLR isolated (n = 32)				ACLR combined (n = 25)				Controls (n = 9)			
	Baseline	Follow-up	Mean change	*P* value	Baseline	Follow-up	Mean change	*P* value	Baseline	Follow-up	Mean change	*P* value
Medial tibia	2043.3 (553.0)	2134.3 (594.4)	91.0 (18.2, 163.7)	0.02*	2245.1 (468.9)	2354.6 (506.2)	109.4 (27.2, 191.7)	0.01*	2663.0 (800.7)	2818.7 (825.6)	155.7 (71.2, 240.1)	0.003*
Lateral tibia	2669.1 (696.0)	2788.3 (724.1)	119.2 (40.6, 197.7)	0.004*	2918.7 (795.7)	3007.5 (835.4)	88.8 (3.0, 174.6)	0.04*	3241.2 (903.7)	3246.2 (963.3)	5.0 (− 125.3, 135.3)	0.93

* Significant difference (*P* < 0.05). Cartilage volume change = follow-up - baseline, thus positive values represent a cartilage volume increase

Cartilage defect scores in each group were unchanged over 2 years (Additional file 2: Table S3). The combined ACLR group showed a decrease in BML scores at the medial tibia (*P* = 0.03) over 2 years. No additional significant within-group changes in BML scores were identified (Additional file 3: Table S4).

### Between-group comparisons

The adjusted annual percentage change in tibial cartilage volume in each group is shown in Table 3. After adjustment for confounders, the isolated ACLR group showed significantly greater annual increase than controls at the lateral tibia (*P* = 0.02, mean difference 3.1, 95% Confidence Interval [CI] 0.5, 5.7%). There were no significant differences between the combined ACLR group and controls. The isolated ACLR group also showed significantly greater increase compared to the combined ACLR group at the lateral tibia (*P* = 0.04, mean difference 2.1, 95% CI 0.1, 4.0%).

**Table 3:** Adjusted annual percentage increase in tibial cartilage volume between the three groups

Site	ACLR isolated (n = 32)	ACLR combined (*n* = 25)	Controls (*n* = 9)	*P* value#
Medial tibia	3.9 (1.5, 6.2)	2.7 (0.0001, 5.4)	2.7 (−1.8, 7.1)	0.78
Lateral tibia	2.7 (1.5, 4.0)^a b^	0.7 (−0.8, 2.1)^b^	−0.4 (−2.7, 2.0)^a^	0.03*

Data presented as mean (95% confidence interval). * Significant difference (*P* < 0.05). # Adjusted for age, gender, BMI, baseline cartilage defect and baseline bone size. Post hoc testing was significantly different for ^a^ isolated ACLR versus controls; ^b^ isolated ACLR versus combined ACLR.

The majority of participants in each group had stable cartilage defects over 2 years (Table 4) and, as such, there were no significant differences between the three groups at any TFJ site.

**Table 4:** Cartilage defect change (baseline to follow-up) between the three groups

Site	Cartilage defects change	ACLR isolated (n = 32)	ACLR combined (n = 25)	Controls (n = 9)	*P* value
Medial tibia	Progression	1 (3%)	0 (0)	0 (0)	1.0
	Stable	31 (97%)	24 (96%)	9 (100%)	1.0
	Regression	0 (0)	1 (4%)	0 (0)	0.52
Medial femoral condyle	Progression	2 (6%)	4 (16%)	0 (0)	0.41
	Stable	27 (84%)	20 (80%)	9 (100%)	0.51
	Regression	3 (9%)	1 (4%)	0 (0)	0.79
Lateral tibia	Progression	2 (6%)	0 (0)	0 (0)	0.63
	Stable	25 (78%)	23 (92%)	9 (100%)	0.27
	Regression	5 (16%)	2 (8%)	0 (0)	0.56
Lateral femoral condyle	Progression	1 (3%)	2 (8%)	0 (0)	0.73
	Stable	31 (97%)	20 (80%)	9 (100%)	0.09
	Regression	0 (0)	3 (12%)	0 (0)	0.10

Data presented as number (%).

As shown in Table 5, diverse temporal changes (i.e., progression, stable, and regression) in BMLs were observed for each group. A significantly greater number of participants in both ACLR groups showed BMLs regression at the lateral tibia compared with the control group (isolated ACLR *P* = 0.01; combined group, *P* = 0.04). There were no differences in BMLs between the three groups at the other sites.

**Table 5:** BML change (baseline to follow-up) between the three groups

Site	BML change	ACLR isolated (n = 32)	ACLR combined (n = 25)	Controls (n = 9)	*P* value
Medial tibia	Progression	9 (28%)	5 (20%)	0 (0)	0.25
	Stable	15 (47%)	9 (36%)	6 (67%)	0.29
	Regression	8 (25%)	11 (44%)	3 (33%)	0.46
Medial femoral condyle	Progression	3 (9%)	3 (12%)	0 (0)	0.85
	Stable	25 (78%)	19 (76%)	9 (100%)	0.33
	Regression	4 (13%)	3 (12%)	0 (0)	0.74
Lateral tibia	Progression	7 (22%)	4 (16%)	0 (0)	0.43
	Stable	11 (34%) ^a^	12 (48%) ^b^	9 (100%) ^a, b^	0.001*
	Regression	14 (44%) ^a^	9 (36%) ^b^	0 (0) ^1 2^	0.04*
Lateral femoral condyle	Progression	4 (13%)	5 (20%)	2 (22%)	0.59
	Stable	23 (72%)	17 (68%)	6 (67%)	0.94
	Regression	5 (16%)	3 (12%)	1 (11%)	1.0

Data presented as number (%). *Significant difference (*P* < 0.05). Post hoc was significantly different for ^a^ isolated ACLR versus controls and ^b^ combined group versus controls (*P* < 0.05).

### Associations between baseline cartilage defect and BML scores and tibial cartilage volume increase in ACLR participants

**Fig. 1:**
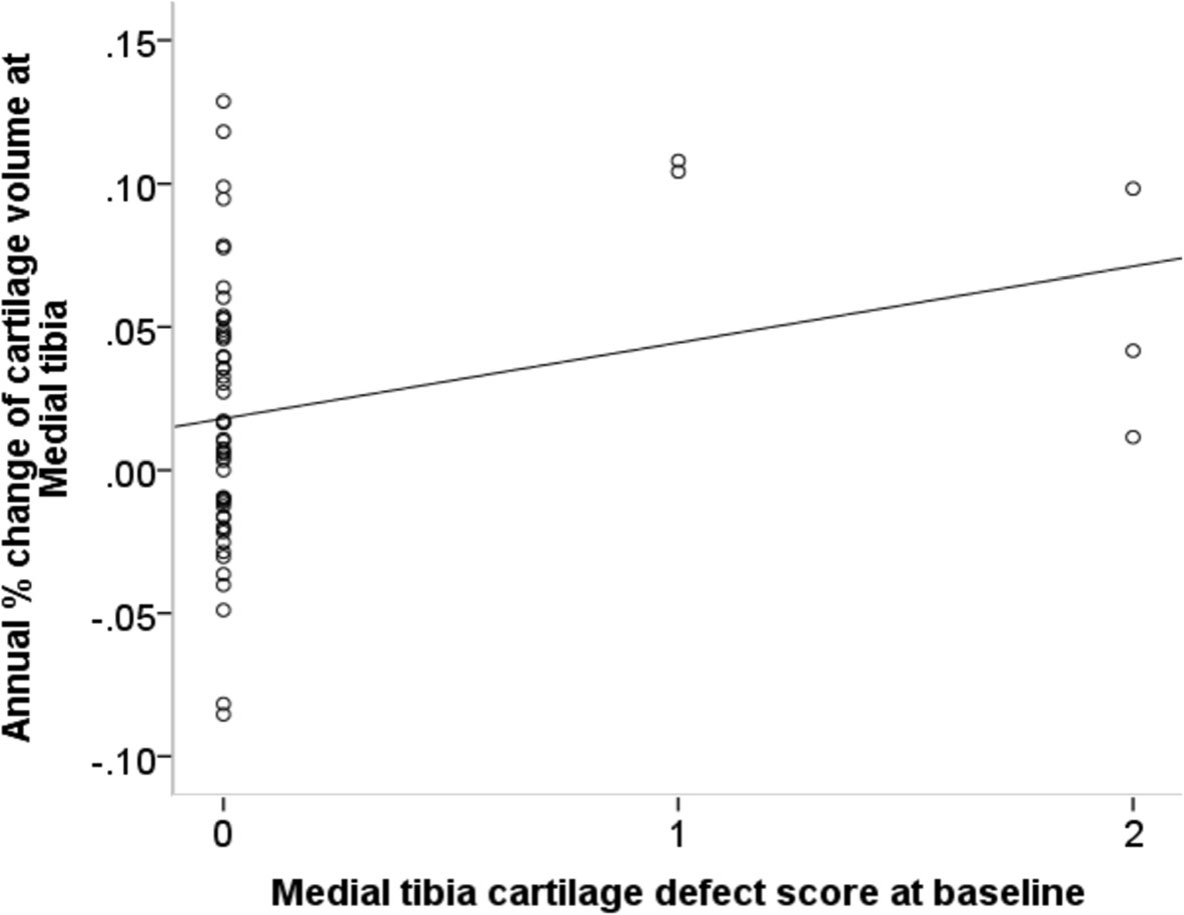
Association between cartilage defect score at baseline and annual percentage increase of cartilage volume at medial tibia before adjustment

**Fig. 2:**
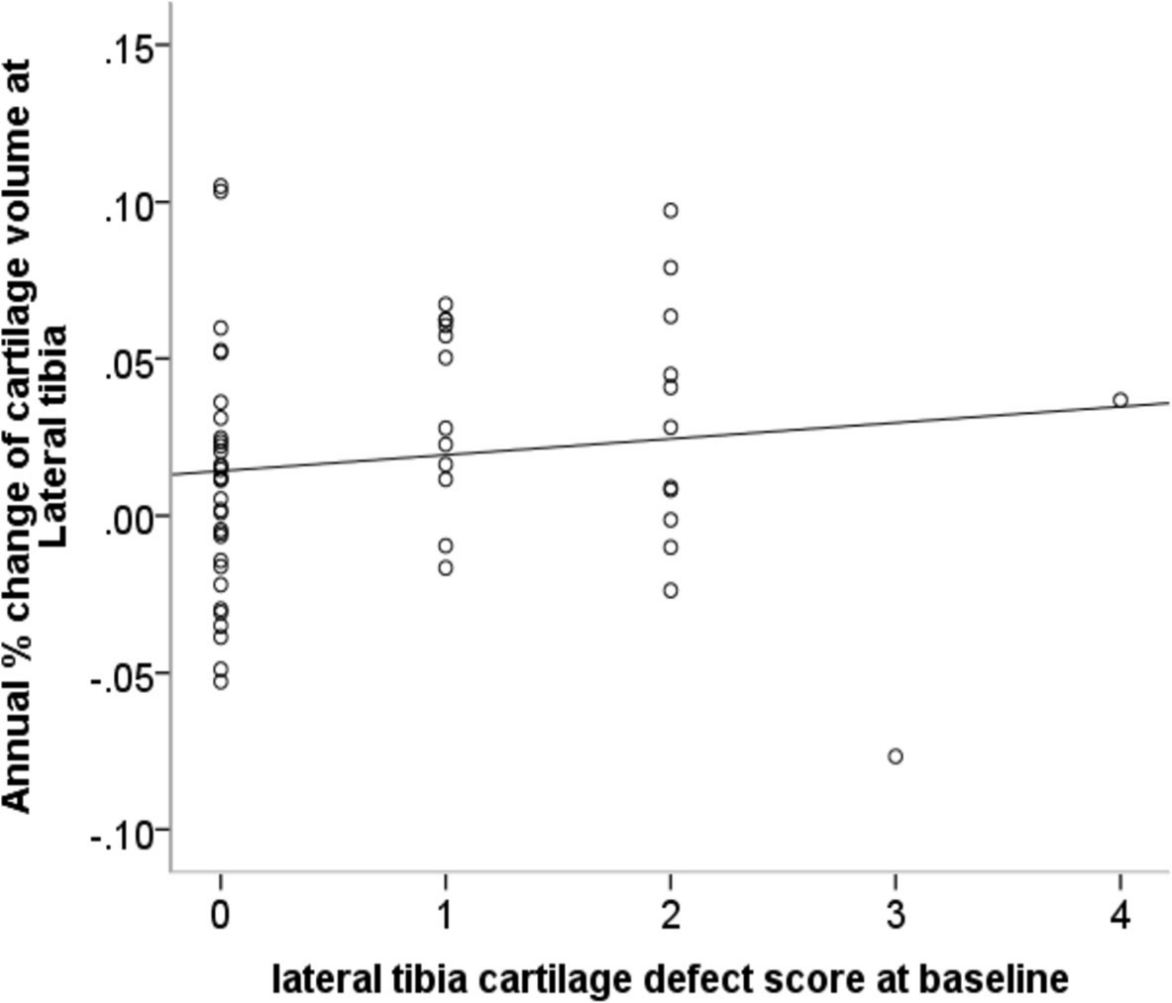
Association between cartilage defect score at baseline and annual percentage increase of cartilage volume at lateral tibia before adjustment

Associations between baseline cartilage defects and BMLs scores with annual tibial cartilage volume percentage change were explored in pooled ACLR participants (*n* = 57). Before adjustment, baseline tibial cartilage defect scores were positively associated with an increase in compartment-specific cartilage volume (medial tibia *P* = 0.02, lateral tibia *P* = 0.01, Table 6, Fig. 1 and Fig. 2). After adjustment for potential confounders, baseline cartilage defects scores in the lateral tibia were positively associated with lateral tibial cartilage volume increase (regression coefficient (B) = 0.02; 95% CI 0.008, 0.032; R^2^ = 0.28; *P* = 0.002).

**Table 6:** Association between baseline tibial cartilage defect, BML scores and annual percentage increase in cartilage volume

	Cartilage Defect				BMLs			
	Univariate regression coefficient (95% CI)	*P* Value	Multivariate regression coefficient (95% CI) ^a^	*P* Value	Univariate regression coefficient (95% CI)	*P* Value	Multivariate regression coefficient (95% CI) ^a^	*P* Value
Medial tibia	0.039 (0.006, 0.071)	0.02*	0.026 (− 0.007, 0.060)	0.12	−0.015 (− 0.025, − 0.005)	0.005*	− 0.017 (− 0.027, − 0.007)	0.001*
Lateral tibia	0.015 (0.003, 0.026)	0.01*	0.02 (0.008, 0.032)	0.002*	−0.004 (− 0.013, 0.004)	0.31	− 0.007(− 0.015, 0.002)	0.11

^a^ Multivariate analysis was adjusted for age, gender, BMI, baseline bone size and presence of meniscal pathology. * Significant difference (*P* < 0.05)

**Fig. 3:**
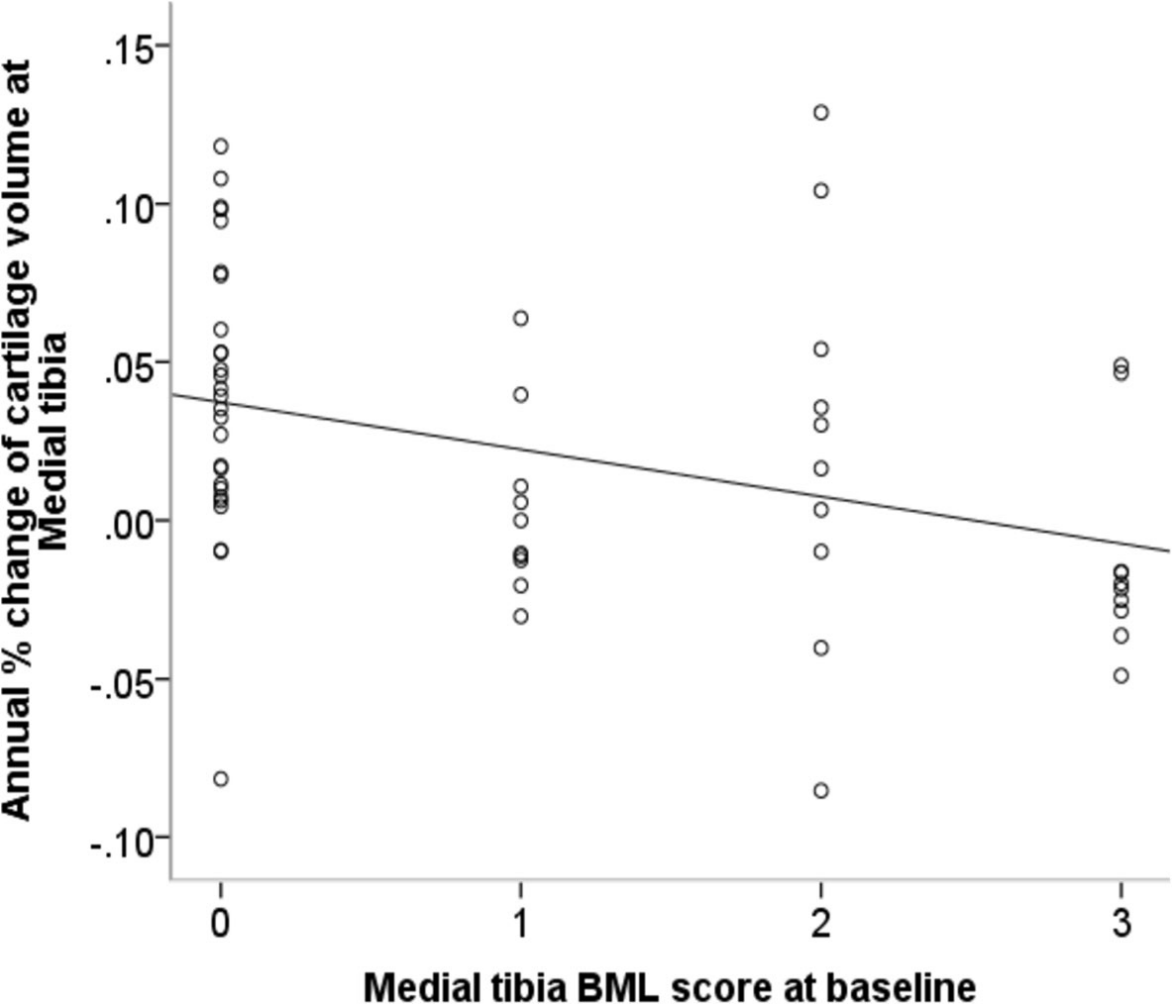
Association between BML score at baseline and annual percentage increase at medial tibia before adjustment

For BMLs, an unadjusted association was only found in the medial tibia where the baseline BML score was negatively associated with cartilage increase (*P* = 0.005, Table 6, Fig. 3). The same negative association in the medial tibia was identified after adjustment for potential confounders (B = -0.017; 95% CI -0.027, − 0.007; R^2^ = 0.59; *P* = 0.001; Table 6).

## Discussion

The primary aim of this study was to investigate the 2-year change in cartilage morphology and BMLs in individuals having undergone ACLR, with and without concomitant meniscal pathology. Temporal response of the different ACLR knee joint structures was mixed, but both ACLR groups showed a significant increase in cartilage volume at the medial and lateral tibia, while controls exhibited an increase only at the medial tibia. The isolated ACLR group exhibited greater increase in lateral tibia cartilage volume compared to both controls and the combined ACLR group. Cartilage defect scores in all groups were unchanged, whilst BMLs regressed in the medial tibia of the combined ACLR group. In the lateral tibia, more ACLR participants (from both groups) showed BML regression compared to controls. Finally, baseline cartilage defects were positively associated with cartilage volume increase in the lateral tibia, while baseline BMLs were negatively correlated with cartilage volume increase in the medial tibia in ACLR knees.

In partial support of H_1_, an overall increase in tibial cartilage volume was observed in both ACLR groups at both medial and lateral tibia over the 2-year period. Several longitudinal studies have also reported increased cartilage volume or thickness from 1 to 5 years following ACLR [12–15]. In contrast to those with established knee OA, where *reductions* in cartilage volume/thickness are characteristic of disease progression [35, 36], increases in cartilage volume or thickness have been reported in early OA populations [16, 17] and precede cartilage loss. Increasing cartilage volume post-ACLR is suggestive of early cartilage degeneration, likely caused by cartilage hypertrophy or cartilage swelling [12]. Cartilage morphology and function are maintained by a delicate balance between swelling properties of the proteoglycans and counteracting collagen tension [37, 38]. In the initial stages of cartilage degeneration, damaged collagen networks do not adequately resist the swelling pressures. As a result, the water content in cartilage increases and cartilage volume increases accordingly [37, 38]. The increased cartilage volume could also reflect accelerated cartilage metabolism in an attempt to repair initial cartilage damage and withstand mechanical load [39, 40]. Irrespective of the underlying mechanism, the increased cartilage volume observed in the ACLR groups represents a disturbance in cartilage homeostasis, which is likely to be related to greater susceptibility to OA in the long-term [12]. Although it was hypothesised that there would be no change in cartilage volume in the control group (H_1_), a significant increase was found at the medial tibia. This increase may represent disturbed cartilage homeostasis in healthy people without knee symptoms, or may be an artefact of participant attrition at follow-up.

Interestingly, the combined ACLR group exhibited less annual percentage cartilage volume increase than the isolated ACLR group (albeit only results for the lateral tibia reached significance). On face value, these results are somewhat counterintuitive as it was hypothesised (H_2_) that the combined group would demonstrate a more pronounced cartilage increase than the isolated group given that concomitant meniscal pathology is a primary contributor to knee OA after ACLR [5, 41], and thus likely a contributor to post-ACLR cartilage swelling. An explanation for the paradoxical findings might relate to the fact that cartilage volume change was measured across the entire cartilage plate. Eckstein et al. (2014) identified simultaneous cartilage thickening and thinning in different sub-regions of the same cartilage plate after ACLR [12]. Therefore, the overall magnitude of cartilage volume change in one cartilage plate depended upon the balance of cartilage thickening and thinning from all sub-regions. The isolated ACLR group experienced a greater cartilage volume increase than the other groups suggesting that on balance, increases were predominant across the cartilage plate sub-regions. In contrast, the combined group may have been undergoing cartilage thinning in several sub-regions because of more advanced cartilage degeneration. Further investigation using region-specific assessment of cartilage morphology is warranted to confirm this theory. At the medial tibia, by contrast, the non-significant finding between the ACLR and control groups could be explained by the fact that cartilage volume also increased in the control group.

Whilst the combined ACLR group had greater prevalence of lateral tibia cartilage defects than the isolated ACLR group at baseline [31] cartilage defect scores generally remained unchanged in the ACLR and control groups over the 2-year follow-up. As such, there were no change-related between-group differences (in contrast to H_2_). This finding is supported by an 11-year longitudinal study by Porter et al. [19] who reported minimal change in TFJ cartilage defects in ACLR knees until degenerative changes accelerated at 5 to 7 years post-surgery.

The BML scores in the combined ACLR group showed a significant improvement in the medial tibia, while they remained unchanged over the two years in the other groups. Specifically, 44% of participants in the combined ACLR group exhibited BML regression. Improvements in medial tibia BMLs in the combined ACLR group, but not the isolated ACL group, may be due to differences in joint unloading between ACLR groups. In established knee OA, higher knee loading contributes to an increased risk of BMLs [42]. It is possible that improvements in BMLs are influenced by loading changes post-ACLR, wheresome evidence actually suggests relative joint unloading; a recent study demonstrated that ACLR patients exhibit lower medial TFJ compartment contact forces in their involved limb compared to non-involved limb during gait and sports-related activities [43]. Whilst the mechanistic pathway by which unloading alters the metabolism of the cartilage-subchondral bone unit leading to a resolution of BMLs is currently unclear, it may be related to changes in intra-osseous hypertension [44].

Previous studies have reported a resolution of post-traumatic BMLs in the lateral knee compartment, particularly in lateral tibia [14, 22–26]. Although we identified a resolution of lateral TFJ compartment BMLs of 44 and 36% of participants in the isolated and combined ACLR groups, respectively, between-ACLR group comparisons failed to reach statistical significance (in contrast to H_2_). Non-significant differences may be attributable to multi-factor mechanisms contributing to BMLs, since trauma, joint loading, and physical activity are all associated with BML change [42, 45]. Furthermore, a lack of statistical power due to a relatively small sample size may have contributed to this non-significant finding. Importantly however, a greater number of participants in both ACLR groups showed regression of lateral tibial BMLs compared with the control group (in support of H_1_).

Higher baseline cartilage defect scores in the lateral tibia of ACLR participants were associated with greater annual percentage tibial cartilage volume increases over the subsequent two years (not medial tibia, in partial support of H_3_). This finding supports the notion that cartilage volume increase represents early cartilage degeneration [12]. For the first time, the current study has demonstrated a quantitative relationship between TFJ cartilage defects and subsequent increases in tibial cartilage volume in ACLR patients. From a mechanistic perspective, cartilage defects alter the normal distribution of loading in the TFJ and thereby, predispose the joint to further degenerative changes [46]. Whilst previous studies have associated severe cartilage defects with OA progression after ACLR [6, 47, 48], the current findings indicate that even mild cartilage defects contribute to disturbed cartilage homeostasis. Clearly, maintenance of cartilage homeostasis is critical in the years following ACLR and there is a need for additional studies to develop more optimal treatment strategies [49]. At the very least, clinicians should take cartilage defects into consideration when counselling patients given the likelihood of long-term cartilage degenerative changes. The lack of association between medial tibia cartilage defects and volume is not surprising given the low prevalence of baseline cartilage defects (13%) compared with the high prevalence in the lateral tibia (60%).

This is the first known study to examine the relationship between BMLs and change in medial tibia cartilage volume in an ACLR cohort. Higher scores for baseline BMLs in the medial tibia were associated with less cartilage volume increase (in contrast to H_3_), suggesting that the presence of large size BMLs in the medial tibia could be a risk factor contributing to subsequent cartilage degeneration in ACLR patients. The effect of BMLs in the medial tibia of our ACLR cohort are analogous to degenerative BMLs in radiographic knee OA patients because BMLs have been associated with cartilage volume loss over two years, particularly in the medial compartment [29, 30, 50]. It may be that BMLs in the medial tibia impede the hypertrophic repair response of the overlying cartilage, given that BMLs are suggested to reduce the stress-dissipating capacity of the cartilage-subchondral bone unit and, could also inhibit nutritional flow from the bone marrow to cartilage [20]. By contrast, no significant associations were identified between BMLs and cartilage volume in the lateral tibia. These findings suggest that the medial and lateral cartilage-subchondral bone units respond quite differently. In accordance with our finding, previous studies have reported that BML size in the lateral TFJ is not associated with cartilage loss in the first 3 years following ACL injury [14, 19].

This study has several strengths. First, the study included a control group that was comparable in age and physical activity levels to the ACLR cohorts. By contrast, other longitudinal studies of this nature have not included a control group to assess the natural history of cartilage morphological change. Second, ACLR participants with and without concomitant meniscal pathology were included. Importantly, the currently exploratory study compared the change in joint morphology between ACLR participants with and without concomitant meniscal pathology over 2 years. By contrast, previous longitudinal studies have focused on joint morphology changes in ACL ruptured [12–14] and ACLR [15] patients - without grouping according to meniscal pathology. This is important given the large differences in the subsequent risk of early-onset knee OA in these groups. Third, this study incorporated a longitudinal design and included a number of measures of joint structure over a 2-year follow-up period.

The study also has limitations. Only 51% of participants returned for follow-up assessment despite considerable effort by the research team to minimise attrition. Importantly however, there was no difference in demographic features of those participants who remained in the study and those lost to follow-up. The sample size at follow-up was relatively moderate, particularly for the combined ACLR and control groups. This reduces the statistical power of the study and increases the likelihood of a Type II error. A sample size of 32 participants provides an effect size of 0.5 to detect cartilage volume change within each group with 80% power and an alpha level of 0.05. With 25 participants, the power is 70%, and with 9 participants, the power decreases to 32%. Given the exploratory nature of the study, no adjustments were made to the alpha level. However, large numbers of statistical tests were performed and this can increase the risk of a Type I error. Future studies with larger sample size and longer follow-up are required to confirm the results of this study.

## Conclusions

The response of the different ACLR knee joint structures was mixed from 2.5 to 4.5 years post-surgery, but likely clarifies several structural adaptations that precede the development of post-traumatic knee OA. Specifically, tibial cartilage hypertrophy, suggestive of altered cartilage homeostasis and early degenerative changes, was apparent in ACLR knees and partly dependent upon meniscal status together with the nature (i.e., cartilage defects or BMLs) and location (i.e., medial or lateral tibia) of the underlying pathology at baseline. Interestingly, cartilage defects and BMLs did not progressively worsen. Indeed, BMLs in the medial tibia of the combined ACLR group actually improved over the follow-up period. Hence, the magnitude and direction of change in MRI-derived joint pathologies following ACLR appear less predictable than previously thought.

## Additional files

Additional file 1: **Table S1.** Characteristics of ACLR participants who completed the study and those who were lost to follow-up. **Table S2.** Characteristics of control participants who completed the study and those who were lost to follow-up. (DOCX 17 kb)

Additional file 2: **Table S3.** Median (IQR) baseline and follow-up cartilage defect score with pre-post Wilcoxon test in each group. (DOCX 16 kb)

Additional file 3: **Table S4.** Median (IQR) baseline and follow-up BML score with pre-post Wilcoxon test in each group. (DOCX 17 kb)

## Data Availability

The datasets used and analysed in the current study are available from the corresponding author on reasonable request.

## Abbreviations

ACL: Anterior cruciate ligament
ACLR: Anterior cruciate ligament reconstruction
ANCOVA: Analysis of covariance
BMI: Body mass index
BML: Bone marrow lesion
CI: Confidence Interval
ICCs: Intra-class correlation coefficients
ICRS: International Cartilage Repair Society
MRI: Magnetic resonance imaging
OA: Osteoarthritis
PD: Proton density
TFJ: Tibiofemoral joint
