# Supplementary material for: Tibiofemoral joint structural change from 2.5 to 4.5 years following ACL reconstruction with and without combined meniscal pathology
Source: BMC Musculoskelet Disord. 2019 Jul 4;20:312. doi: 10.1186/s12891-019-2687-9 (PMC6610973; doi:10.1186/s12891-019-2687-9)
Supplement: Supplementary file 1 — Table S1. Characteristics of ACLR participants who completed the study and those who were lost to follow-up. Table S2. Characteristics of control participants who completed the study and those who were lost to follow-up. (DOCX 17 kb) [file 12891_2019_2687_MOESM1_ESM.docx]

Table S1 Characteristics of ACLR participants who completed the study and those who were lost to follow-up

|  | **Completed study (n = 57)** | **Lost to follow-up (n = 43)** | **P value** |
| --- | --- | --- | --- |
| Age at baseline (yr) | 30.7 (± 6.7) | 29.4 (± 6.3) | 0.34 |
| Gender, male (%) | 37 (65%) | 29 (67%) | 0.83 |
| BMI (kg/m^2^) | 25.6 (± 3.6) | 24.8 (± 3.6) | 0.28 |
| Meniscal pathology, n (%) | 25 (44%) | 13 (30%) | 0.21 |
| Sports activity level at baseline | 85 (80, 95) | 88 (79, 95) | 0.23 |

BMI, body mass index. Parametric data presented as mean (± standard deviation), and sports activity levels presented as median (interquartile range).

Table S2 Characteristics of control participants who completed the study and those who were lost to follow-up

|  | **Completed study (n = 9)** | **Lost to follow-up (n = 21)** | ***P* value** |
| --- | --- | --- | --- |
| Age at baseline (yr) | 28.3 (± 4.0) | 28.4 (± 5.7) | 0.95 |
| Gender, male (%) | 8 (89%) | 11 (52%) | 0.07 |
| BMI (kg/m^2^) | 24.6 (± 3.8) | 22.9 (± 3.0) | 0.19 |
| Sports activity level at baseline | 78 (85, 88) | 90 (75, 100) | 0.51 |

BMI, body mass index. Parametric data presented as mean (± standard deviation), and sports activity levels presented as median (interquartile range).
