# Supplementary material for: Tibiofemoral joint structural change from 2.5 to 4.5 years following ACL reconstruction with and without combined meniscal pathology
Source: BMC Musculoskelet Disord. 2019 Jul 4;20:312. doi: 10.1186/s12891-019-2687-9 (PMC6610973; doi:10.1186/s12891-019-2687-9)
Supplement: Supplementary file 2 — Table S3. Median (IQR) baseline and follow-up cartilage defect score with pre-post Wilcoxon test in each group. (DOCX 16 kb) [file 12891_2019_2687_MOESM2_ESM.docx]

Table S3 Median (IQR) baseline and follow-up cartilage defect score with pre-post Wilcoxon test in each group

| **Site** | **ACLR isolated (n = 32)** | | | **ACLR combined (n = 25)** | | | **Controls (n = 9)** | | |
| --- | --- | --- | --- | --- | --- | --- | --- | --- | --- |
|  | Baseline | Follow-up | P value | Baseline | Follow-up | P value | Baseline | Follow-up | P value |
| Medial tibia | 0 (0, 0) | 0 (0, 0) | 0.32 | 0 (0, 0) | 0 (0, 0) | 0.32 | 0 (0, 1) | 0 (0, 1) | 1.0 |
| Medial femoral condyle | 0 (0, 0) | 0 (0, 0) | 0.58 | 0 (0, 0.5) | 0 (0, 2) | 0.10 | 0 (0, 0) | 0 (0, 0) | 1.0 |
| Lateral tibia | 0 (0, 1) | 0 (0, 1) | 0.53 | 0 (0, 2) | 0 (0, 2) | 0.18 | 1 (0, 1) | 1 (0, 1) | 1.0 |
| Lateral femoral condyle | 0 (0, 2) | 0 (0, 2) | 0.32 | 1 (0, 2) | 0 (0, 2) | 0.89 | 0 (0, 0) | 0 (0, 0) | 1.0 |
