# Supplementary material for: Tibiofemoral joint structural change from 2.5 to 4.5 years following ACL reconstruction with and without combined meniscal pathology
Source: BMC Musculoskelet Disord. 2019 Jul 4;20:312. doi: 10.1186/s12891-019-2687-9 (PMC6610973; doi:10.1186/s12891-019-2687-9)
Supplement: Supplementary file 3 — Table S4. Median (IQR) baseline and follow-up BML score with pre-post Wilcoxon test in each group. (DOCX 17 kb) [file 12891_2019_2687_MOESM3_ESM.docx]

Table S4 Median (IQR) baseline and follow-up BML score with pre-post Wilcoxon test in each group

| **Site** | **ACLR isolated (n = 32)** | | | **ACLR combined (n = 25)** | | | | **Controls (n = 9)** | | |
| --- | --- | --- | --- | --- | --- | --- | --- | --- | --- | --- |
|  | Baseline | Follow-up | *P* value | Baseline | Follow-up | *P* value | Baseline | | Follow-up | *P* value |
| Medial tibia | 1 (0, 2) | 0 (0, 2) | 1.0 | 1 (0, 2) | 0 (0, 1) | 0.03* | 0 (0, 1) | | 0 (0, 0) | 0.1 |
| Medial femoral condyle | 0 (0, 0) | 0 (0, 0) | 0.93 | 0 (0, 0) | 0 (0, 0) | 0.67 | 0 (0, 0) | | 0 (0, 0) | 1.0 |
| Lateral tibia | 1 (0, 2) | 0 (0, 1.5) | 0.06 | 0 (0, 2) | 0 (0, 0.25) | 0.09 | 0 (0, 0) | | 0 (0, 0) | 1.0 |
| Lateral femoral condyle | 0 (0, 0) | 0 (0, 0) | 0.87 | 0 (0, 0) | 0 (0, 0) | 0.40 | 0 (0, 1) | | 0 (0, 1.5) | 0.79 |

* Significant difference (*P* < 0.05).
